# Linking demographic processes and foraging ecology in wandering albatross—Conservation implications

**DOI:** 10.1111/1365-2656.12817

**Published:** 2018-03-30

**Authors:** Henri Weimerskirch

**Affiliations:** ^1^ Centre d'Etudes Biologiques de Chizé UMR 7372, CNRS/Université de La Rochelle Villiers‐en‐Bois France

**Keywords:** capture–mark–recapture, *Diomedea exulans*, population dynamics, vital rates

## Abstract

1. Population dynamics and foraging ecology are two fields of the population ecology that are generally studied separately. Yet, foraging determines allocation processes and therefore demography. Studies on wandering albatrosses *Diomedea exulans* over the past 50 years have contributed to better understand the links between population dynamics and foraging ecology. This article reviews how these two facets of population ecology have been combined to better understand ecological processes, but also have contributed fundamentally for the conservation of this long‐lived threatened species.
2. Wandering albatross research has combined a 50‐year long‐term study of marked individuals with two decades of tracking studies that have been initiated on this species, favoured by its large size and tameness.
3. At all stages of their life history, the body mass of individuals plays a central role in allocation processes, in particular in influencing adult and juvenile survival, decisions to recruit into the population or to invest into provisioning the offspring or into maintenance.
4. Strong age‐related variations in demographic parameters are observed and are linked to age‐related differences in foraging distribution and efficiency. Marked sex‐specific differences in foraging distribution, foraging efficiency and changes in mass over lifetime are directly related to the strong sex‐specific investment in breeding and survival trajectories of the two sexes, with body mass playing a pivotal role especially in males.
5. Long‐term study has allowed determining the sex‐specific and age‐specific demographic causes of population decline, and the tracking studies have been able to derive where and how these impacts occur, in particular the role of long‐line fisheries.

## 1 INTRODUCTION

**Figure 1:**
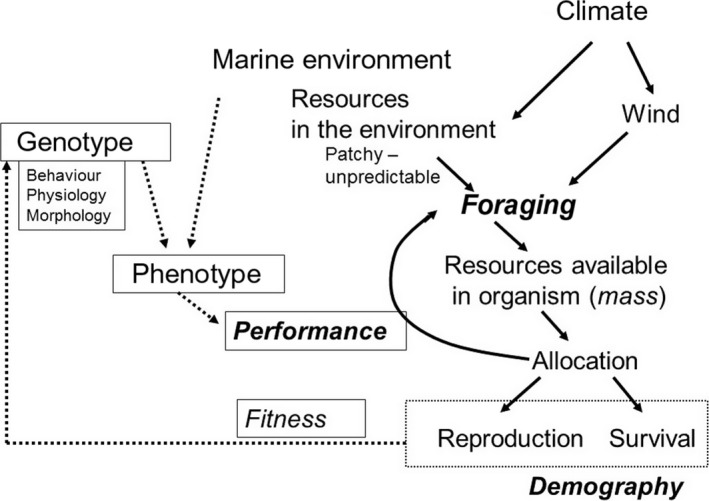
Conceptual framework showing the links between climate and demography through foraging and allocation processes

Changes in population sizes results from demographic processes, and the associated evolutionary processes, take place over long periods of time, especially in long‐lived animals. Thus, long‐term individual‐based studies are necessary to address fundamental questions about these processes (Clutton‐Brock & Sheldon, 2010). In particular, long‐term studies allow measurements on an annual basis of demographic parameters at the individual level to understand the reasons for variation in population size. But the demographic traits themselves result from complex allocation processes (Stearns, 1992), trade‐offs between reproduction and survival, that depend entirely on the foraging performance of individuals (Figure 1) and thus the resource acquisition (VanNoordwijk & DeJong, 1986). The ability of an individual to extract resources from the environment determines the amount of energy available to an individual to be expended in fitness‐related activities, such as self‐maintenance and reproduction, and the relative level of between‐individual variation in resource acquisition determines whether we can observe the trade‐offs (VanNoordwijk & DeJong, 1986). Foraging efficiency is known to be a major determinant of individual fitness (Stephens, Brown, & Ydenberg, 2007). Thus, foraging and the allocation of resources during reproduction should be considered under the conceptual framework of life‐history theory (Stearns, 1992), and foraging effort could be regarded as the result of an allocation decision (Boggs, 1992). In this context, evidence is accumulating about the role of the animal physiological state, for example body condition, in these allocation decisions (McNamara & Houston, 1996), underlining the interest of recording information on the condition or mass of individuals in long‐term studies (Ozgul et al., 2010). Being able to couple long‐term demographic studies, mass and foraging behaviour at the individual level make it possible to investigate the links between foraging and survival and reproduction, and ultimately the links between the environment and the demography of populations (Figure 1).

Long‐term studies started in the late 1940s and 1950s on several species of birds, such as great tits, kittiwakes or fulmars, and have been the catalysts for many new long‐term studies in the following decades (Clutton‐Brock & Sheldon, 2010). Increasingly, they have played a central role in research in ecology. In the earlier years of long‐term studies, studying foraging behaviour in the wild was not an easy task and difficult to perform at the individual level. Foraging studies were mainly possible in controlled conditions or in the wild were based on observations of non‐marked individuals. It is only since the late 1970s that VHF (very high frequency) telemetry allowed the tracking of individuals in their environment, and in the late 1980s that satellite telemetry emerged as a tracking method (Jouventin & Weimerskirch, 1990), available first on large animals, and progressively with the miniaturization of electronics to smaller and smaller animals. Today, not only it is possible to track remotely and accurately animals in their environment, but also to record many other parameters such as activity, heart rate through methods grouped as bio‐logging (Ropert‐Coudert & Wilson, 2005) and thus to estimate foraging effort. Applying these methodologies to animals from long‐term studies whose age, pedigree or lifetime reproductive success is recorded has opened the possibility to address questions on the links between foraging and demographic traits.

The wandering albatross *Diomedea exulans* has been the animal model in an effort to link demographic traits and foraging behaviour. In this synthesis, I summarize the past 50 years of wandering albatross research on the Crozet Islands that was conducted on two parallel fronts of research that have converged over the past 20 years, demography and foraging ecology. The aims of this review are to (1) briefly describe wandering albatross biology, (2) provide a historical overview of the wandering albatross research from late 1950s, (3) examine how foraging studies have been able to apprehend the age‐related and sex‐specific demography and (4) illustrate how the combination of these two fields of ecological research have been critical for the conservation of seabirds.

## 2 SHORT HISTORY OF WANDERING ALBATROSS RESEARCH

On the Crozet Islands, south‐western Indian Ocean, studies on the wandering albatross started in 1959 with the banding of most breeding birds on one of the large island of the archipelago, Possession Island (52°E, 46°S). Members of the expedition led by Captain Tilman on sailing boat Mischief banded 200 albatrosses with bands provided by the California Wildlife Society. At the same time, Lance Tickell was carrying out a major study on the breeding biology of the species at South Georgia (Tickell, 1968). At Crozet, Jean‐Louis Mougin, who worked with George Dunnet on his starting long‐term study of fulmars, and Jean Prevost from the Paris National Museum set up a demographic study from 1965 on the model of the fulmar mark–recapture protocol, using as a basis the 1959 bandings. Since then, every year wandering albatrosses have been banded as fledglings or adults, and their status (visitor, non‐breeding, breeding etc.….), breeding success and mate identity recorded, and the nest located. The long‐term monitoring programme was taken over from the early 1980s by CNRS researchers and then managed by the CNRS Chizé laboratory as part of a programme funded by the French Polar Institute led by Pierre Jouventin and later myself. With the continuous monitoring programme since 1965, today each wandering albatross from Possession Islands is identified by a metal band, and its age, as well as its pedigree is known. Mass and measurements are taken every year on large sample sizes of adults and fledglings. In addition, since 10 years, we have been studying personalities in our population, measured as a gradient between shy and bold birds reacting to human approach (Patrick, Charmantier, & Weimerskirch, 2013). A total of 14,920 individuals were banded for the Possession Island population, with more than 53,647 capture–recapture histories recorded over 56 years.

Because of their large size, tameness and easy access to their colonies on Crozet, wandering albatrosses have been the animal model on which many new bio‐logging techniques have been experimented first. In 1989, the first successful satellite tracking of a bird was carried out on wandering albatrosses from Crozet (Jouventin & Weimerskirch, 1990), and studies were continued on an annual basis for years with Argos transmitters. In late 1990s—early 2000s, wandering albatrosses were the first species on which GPS (Weimerskirch, Bonadonna et al., 2002) or geolocators measuring light level (Weimerskirch & Wilson, 2000) were tested. Geolocators, albeit providing low accuracy locations, allow the tracking over years of large number of individuals because of their low cost and extremely light weight (1–2 g). As subject of many new advances in foraging studies, such as prey capture recording (Weimerskirch & Wilson, 1992; Wilson, Cooper, & Plötz, 1992), heart rate recording (Weimerskirch, Guionnet, Martin, Shaffer, & Costa, 2000; Weimerskirch, Shaffer et al., 2002) or radar detectors (Weimerskirch, Filippi, Collet, Waugh, & Patrick, 2017), the species has become an animal model for which long‐term demographic studies could be combined with tracking studies, with more than 1,000 individual deployments on the species at Possession Island only.

## 3 WANDERING ALBATROSS BIOLOGY AND POPULATION DYNAMICS

The wandering albatross is a very large seabird (8–12 kg, up to 3.5 m in wingspan) breeding on sub‐Antarctic islands of the Southern Ocean (Photograph 1). It is a very long‐lived animal living more than 60 years of age. The species is sexually dimorphic, males being 20% larger and heavier than females, males weigh 8–12 kg, compared to 7–9 kg in females. The breeding cycle lasts an entire year with nest building starting in December, laying of the single egg in early January. The single large egg is incubated alternatively by the male and female for bouts of 10 days on average (Weimerskirch, 1995), and hatching occurs in mid‐March after an incubation period of 80 days (Tickell, 1968). After 1 month of brooding by both parents alternatively, the chick is left alone on the nest and fed infrequently by both parents so that growth is extremely slow, attaining a peak weight in September, when parents progressively decrease the number of visits to the chick, and then leave it alone on the nest (Weimerskirch & Lys, 2000). In November, the chick has lost 20%–50% of its peak weight and is almost abandoned by its parents. Chicks fledge in November, after 8 months of parental care, one of the longest periods of parental care in birds. They leave the nest independently from their parents, spend several years at sea continuously before visiting their birth place at the age of 3–7 years, and breeding at an average age of 10 years (range 6–15)(Weimerskirch, Brothers, & Jouventin, 1997). After the 1‐year‐long breeding season, parents generally take a sabbatical year when they stay permanently at sea before returning the next breeding season (Tickell, 1968), although some individuals, especially females, are able to breed again the next year (Barbraud & Weimerskirch 2012).

**Photograph 1:**
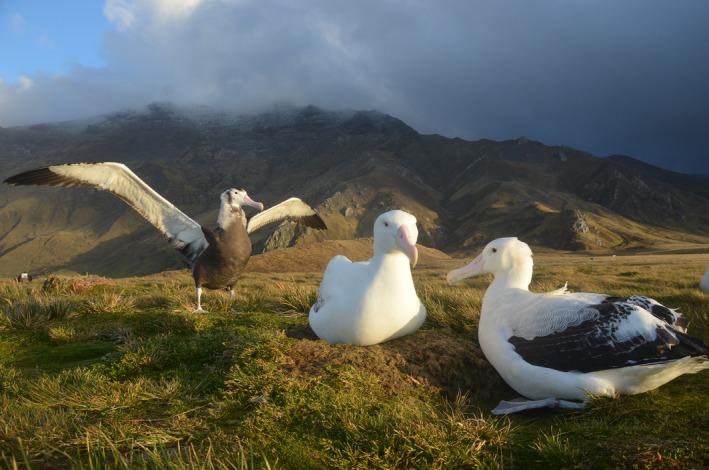
A pair of adult wandering albatrosses on their nest, with a juvenile individual practising flight in the back ground (Photograph H. Weimerskirch) [Colour figure can be viewed at http://wileyonlinelibrary.com]

Wandering albatrosses are well known for using a dynamics soaring flight whereby they use the wind as an energy source (Pennycuick 1982), and studies measuring heart rate have shown that when in flight birds have metabolic rates close to resting (Weimerskirch, Guionnet et al., 2000). The wind is therefore a major determinant of the foraging efficiency (Figure 1). During breeding, birds cover extensive distances to search for prey, mainly squids (Cherel, Xavier, de Grissac, Trouvé, & Weimerskirch, 2017), that are dispersed, and found on average every 64 km (Weimerskirch, Gault, & Cherel, 2005; Weimerskirch, Pinaud, Pawlowski, & Bost, 2007). The search strategy is to cover extensive distances at low costs while searching for prey dispersed over the huge surface of ocean.

When the season ends with the fledging of the chick, parents disperse to sea for a sabbatical year, staying permanently at sea. Satellite tracking and geolocators show that the behaviour is very variable between individuals, from purely sedentary behaviour where birds remain in the western Indian Ocean in a radius of 3–4,000 km around the nesting grounds, to migratory behaviour (Weimerskirch, Delord, Guitteaud, Phillips, & Pinet, 2015). Migratory birds either migrate back and forth between Crozet and various sectors off Australia, using the same sector every second year or do a single, double or triple circumpolar navigation, always with an eastward movement following the westerlies, and staging in one, two or three oceanic sectors off eastern New Zealand, Chili or Argentina (Weimerskirch et al., 2015). These specific sectors are used every second year by the same individual, throughout its lifetime (Weimerskirch, Jouventin, Mougin, Stahl, & VanBeveren, 1985; Weimerskirch & Wilson, 2000). Moult occurs in these sectors, and the extent of moult of the flight feathers depends on the length of the sabbatical period (Weimerskirch, 1991). Males renew more feathers than females. First time breeders have less new feathers than other breeders, suggesting that moult may be a significant constraint in the life history of the species.

## 4 WANDERING ALBATROSS SEX‐ AND AGE‐RELATED DEMOGRAPHY AND FORAGING ECOLOGY, AND THE ROLE OF MASS

**Figure 2:**
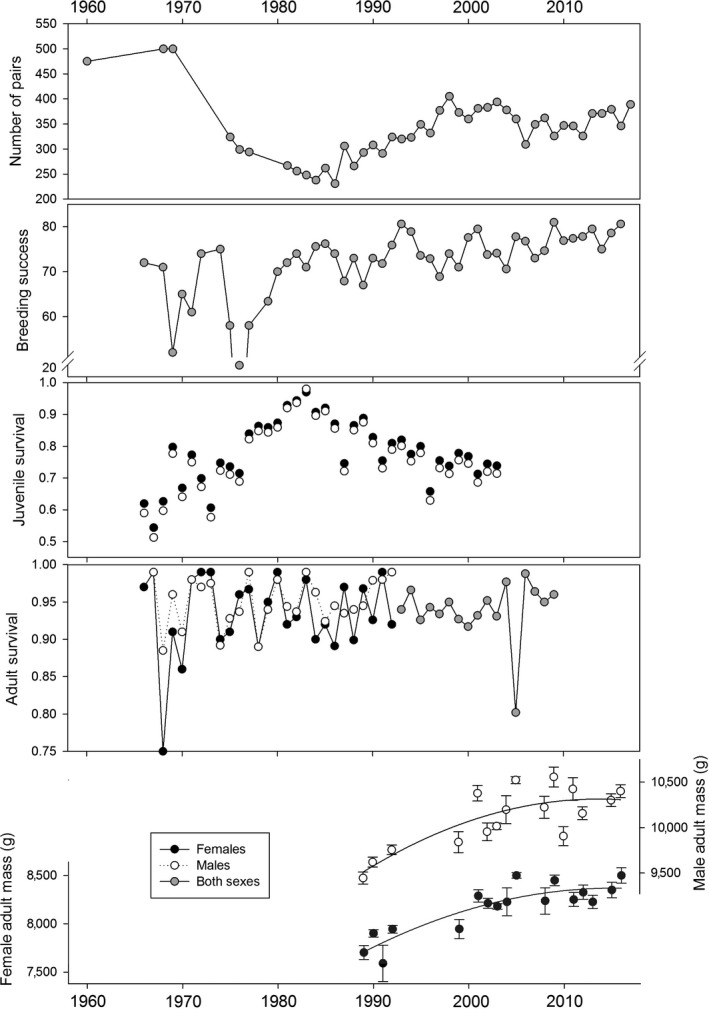
Changes over the study period in the population size, breeding success, adult and juvenile annual survival, and in the mass of breeding males and females

Over the past 50 years, important changes occurred in the demography of the Crozet wandering albatross population (Figure 2). After a period of stability in the 1960s, the number of pairs breeding every year crashed abruptly in the 1970s, and increased from the mid‐1980s to peak at the end of the 1990s, then declined slightly (Figure 2). Over the entire period breeding success increased to attain high values of 80% of eggs producing fledglings leaving the colony. Annual juvenile survival increased until the mid‐1980s and then declined afterwards, while adult survival was high through the period, apart for low values in the 1970s and in 2005. The mass of breeding males and females increased by almost one kilogram over the past 25 years (Figure 2). All these changes have been the results of several factors affecting the males and females differently and find their roots in the strong age‐ and sex‐specific differences in the foraging ecology of the different life stages of the population.

**Figure 3:**
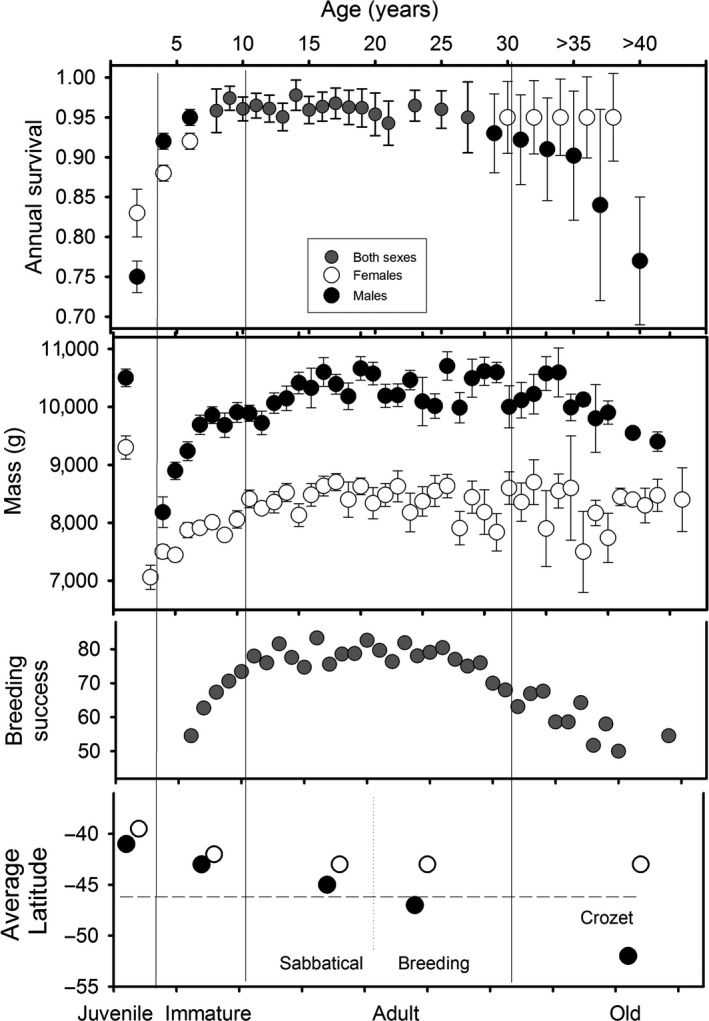
Changes over age in adult survival, mass, breeding success and latitudinal distribution at sea of males and females wandering albatross

Foraging behaviour, the set of processes by which organisms acquire energy and nutrients is considered to play a key role in shaping patterns of age‐specific reproduction in the wild (Forslund & Pärt, 1995). Age, sex and foraging ability interact in shaping ageing patterns in natural conditions. Therefore, we are in a position to examine how the interplay between condition, foraging abilities and demography operates throughout the lifetime of males and females, in particular age‐related changes in mass, breeding success and survival (Figure 3). In wandering albatrosses, age strongly affects survival, reproductive performance, mass and foraging performance (Figure 3), and age affects males and females differently.

### 4.1 Early life and recruitment

The early life of animals is generally a poorly known period, especially the juvenile phase when young animals disperse and are generally affected by a high mortality. Yet, in long‐lived species, younger age classes represent up to half of the total population and variations in vital rates of younger age classes are likely to have a high influence on the population dynamics and the rate of evolutionary change in long‐lived species (Cam & Aubry, 2011; Saether et al., 2013). In addition, there is increasing evidence that conditions experienced in early life may have long‐term individual fitness consequences with important demographic and evolutionary effects (Gaillard, Festa‐Bianchet, Yoccoz, Loison, & Toïgo, 2000). After fledging, juvenile wandering albatrosses disperse widely to the north of the species range, in subtropical waters (Weimerskirch, Akesson, & Pinaud, 2006). Previous studies in mammals and birds suggested that most of the mortality between fledging and recruitment occurs during the first months of life (Gaillard et al., 2000). This pattern has been linked to progressive improvement of foraging skills in early life (Marchetti & Price, 1989). In the wandering albatross, learning of basic foraging skills allowing immediate survival takes place within a few months after fledging. Foraging movement capacities become similar to those of adults within a few months after fledging (Riotte‐Lambert & Weimerskirch, 2013). Individuals are also able during this short period to use efficiently wind conditions and detect environmental gradients (De Grissac, Bartumeus, Cox, & Weimerskirch, 2017). Juvenile survival over the first 2 years of life is 0.64, which is one of the highest values of early‐life survival estimated for a bird species, but still lower than annual adult survival (Figure 3). Males have a higher mortality rate at this early stage than females. Male wandering albatrosses which are structurally larger and heavier than females could be more sensitive to starvation during this critical step of independence due to their higher food requirements for self‐maintenance. Juvenile male survival is higher for individuals in better condition at peak mass during growth (Cornioley, Jenouvrier, Börger, Weimerskirch, & Ozgul, 2017). Males of larger structural size at fledging are those that better survive, whereas females in better body condition survive better (Weimerskirch, Barbraud, & Lys, 2000). Early‐life survival was also highly variable across years although the sensitivity of young birds to environmental variability decreased with age (Fay, Weimerskirch, Delord, & Barbraud, 2015). In addition, early‐life survival was more affected by natal environmental conditions (i.e., those that the parents encountered during their foraging trips to provision the chicks) than by climatic conditions encountered during the first year of life at sea when young animals disperse over large oceanic sectors (Fay et al., 2015). This high susceptibility of natal condition is also observed in other long‐lived species (Gaillard et al., 2000; Reid, Bignal, Bignal, McCracken, & Monaghan, 2003).

After the two‐first years at sea, the sex difference in survival is reversed (Fay et al., 2015), and males have a higher survival than females (Figure 3). Immature birds show sex‐specific distributions at sea with male wandering albatrosses from Possession Island moving more to the east of the southern Indian Ocean than females (Weimerskirch et al., 2014). Thus, environmental conditions experienced by immature individuals could be different between sexes and females may experience less favourable climatic and trophic conditions than males. This period of immaturity is a period when mass increases progressively (Figure 3) and when individuals settle in their future habitat used during sabbatical years when adults. There, they have to moult, and in immatures the extent of moult is related to body mass, in males only (Weimerskirch, 1991), suggesting strong energetic constraints. But at the same time, immatures aged 3–7 years are also visiting the breeding grounds for the first time and then visit regularly the colony: it is the period when young birds are performing their spectacular displays (Photograph 2) used during pair formation. At this time, they alternate short stay on land of displaying in search of a partner with foraging trips at sea similar to those of breeders (Riotte‐Lambert & Weimerskirch, 2013). This period of transition is probably when immature individuals progressively acquire the foraging skills necessary to be an efficient breeder operating as a central place forager from the future colony.

**Photograph 2:**
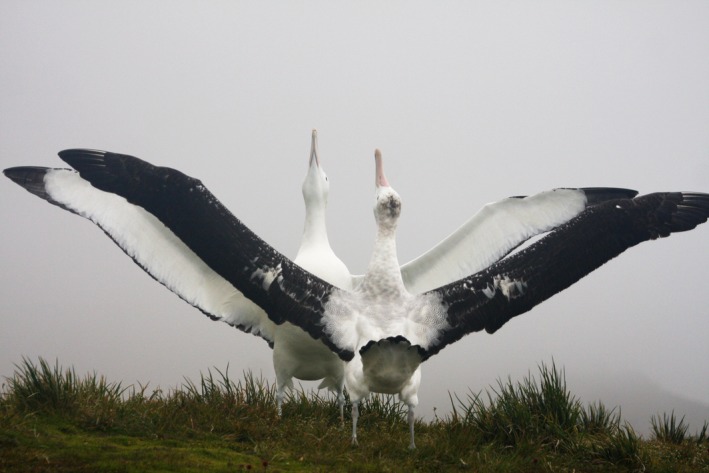
A pair of young wandering albatross in the spectacular “ecstatic display” used during pair formation (Photograph Aurélien Prudor) [Colour figure can be viewed at http://wileyonlinelibrary.com]

### 4.2 Recruitment

This transition phase between first return on land and first reproduction lasts several years, a long period critical for attaining sufficient foraging skills. In the wandering albatross, the onset of breeding depends entirely on mass (Box 1) and is not dependent on age per se. Indeed, wandering albatrosses are sexually mature at age 6 years (Hector, Pickering, Croxall, & Follett, 1990), and some breed at this age in our population, but most individuals recruit several years later. Recruitment depends mainly on the ability of individuals to reach a mass threshold from which they can breed (Weimerskirch, 1992). This threshold is around 8 kg for females and 9.4 kg for males and is attained after several years of immaturity. The ability to attain this minimum mass threshold depends on the foraging efficiency during the phase of transition where birds have to be central place foragers and learn how to forage not only for themselves, but also to store additional energy that will be expended in reproduction. Age at first reproduction is a key demographic parameter that is probably under high selective pressure, but is surprisingly highly variable in the wandering albatross, from 6 to 15 years, and is negatively related to both reproductive and survival adult performances, suggesting that individual quality is an important factor to explain variations in the age at first reproduction (Fay, Barbraud, Delord, & Weimerskirch, 2016).

BOX 1 **Summary of how different body mass during the life‐history stages of the wandering albatross affect vital rates**

1

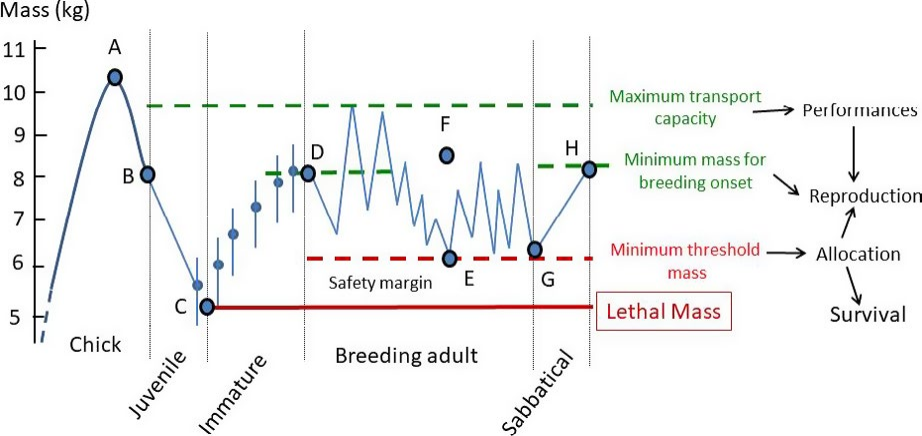

**Table:** 

	Mass	Influence on:	Sex
A	Peak mass during chick growth	Probability of survival during 1st year at sea	M
B	Mass at fledging	Survival until recruitment	M
C	Lethal mass	Death probability	MF
D	Minimum mass for breeding onset	Recruitment probability	MF
E	Minimum threshold mass	Allocation of resources to chick or self‐feeding	MF
F	Working mass	Foraging efficiency	MF
G	Mass at end of breeding season	Moult extent	MF
H	Mass at end of sabbatical year	Breeding probability during next breeding season	MF

### 4.3 Adult stage

When they have attained adulthood, wandering albatrosses have high survival rates, similar in males and females. When breeding, the incubation period is when most failures occur. At this time wind speed increases bird ground speed, which in turn reduces trip duration. Wandering albatrosses forage until their energy requirements is fulfilled, but trip duration does not affect mass gain and lighter individuals gained more mass, suggesting that wandering albatrosses adjust energy requirement to their body condition (Cornioley, Börger, Ozgul, & Weimerskirch, 2016). The mass of males increased breeding success and their survival increased with increasing mass (Cornioley et al., 2017). During the long chick‐rearing period during winter, to provision the chick, wandering albatrosses use a twofold strategy whereby they alternate (1) short trips close to the colony (at hundreds of kilometres) to provide food regularly to the chick, but during these trips where birds do not use optimally the wind conditions, they lose mass; with (2) long trips at thousands of kilometres when adults feed mainly for themselves with a high efficiency using wind conditions optimally, but at the expense of reducing feeding frequency to the chick (Weimerskirch, Barbraud et al., 2000). This strategy is the result of a trade‐off between self‐feeding (survival) and chick provisioning (reproduction) and is under the control of the body mass of the parent (Weimerskirch, Barbraud et al., 2000) (Figure 1, Box 1). When adult mass is good, above a threshold value, the adult provisions the chick, but loses condition until mass falls below the threshold than starts a long trip to restore its condition (Weimerskirch, 1999). The mass is again of utmost importance for decisions regarding breeding investment, as it was for the recruitment into the population and for decisions to breed or not at the start of every breeding season (Weimerskirch, 1992; Box 1). When they provision their chick, the mass of males affects the mass of sons, but not of daughters (Cornioley et al., 2017) giving evidence of higher investment of fathers in sons, but not in daughters with increasing father mass. This strategy is in line with the current theory stating that long‐lived seabirds should adjust their reproductive performance to both their own body conditions and the need of their offspring (Erikstad, Fauchald, Tveraa, & Steen, 1998). Thus, both parents provide more food to sons than to daughters and parents adjust meal size to male chick needs (Weimerskirch, Barbraud et al., 2000). As a consequence, two life‐history outcomes, longevity and lifetime reproductive output, were higher for males that were heavy as chicks compared to initially lighter ones (Cornioley et al., 2017). These results suggest that a higher investment in sons by fathers can increase their inclusive fitness, which is not the case for daughters. Again these results highlight the sex‐specific differences in the effect of body mass on the life history of this monogamous species with bi‐parental care (Cornioley et al., 2017).

When the breeding season ends, both parents disperse in the Southern Ocean to take a sabbatical year, remaining either in the Southern Indian Ocean, or migrating in various sites in the Southern Ocean. However, a small proportion of individuals is able to breed immediately after a successful breeding (Barbraud & Weimerskirch, 2012). Males are more likely to have a long range migratory behaviour, compared to females that are mainly sedentary. This difference in migratory behaviour has strong implications for the fitness of individuals, as almost exclusively females are able to breed annually (Weimerskirch et al., 2015). As partners are extremely faithful during their lifetime, with very low divorce rate (Jouventin, Lequette, & Dobson, 1999), when a female breed during two consecutive seasons and her mate takes a sabbatical year, she changes temporarily of partner (Weimerskirch et al., 2015).

### 4.4 Senescence

Wandering albatrosses have demographic characteristics that make them interesting models for the study of ageing. Being very long‐lived, they can attain more than 60 years, we have in our population individuals aged “more than” 57 years, because they were banded as adults of unknown age 50 years ago. We will need a further 10 or 20 years of monitoring to be able to determine the maximum age they can attain, probably 60–70 years. The ageing pattern especially appearance of senescence nicely falls within the primate continuum of ageing, between chimpanzee and human (Bronikowski et al., 2011), with the same tendencies for males to have shorter life spans and higher mortality at old ages than females (Pardo, Barbraud, & Weimerskirch, 2013), a sex ratio at birth biased systematically towards males (Weimerskirch, Lallemand, & Martin, 2005), suggesting that mortality patterns in long‐lived species are shaped by local selective forces rather than phylogeny (Forslund & Pärt, 1995). Of course, they differ from primates and from other long‐lived marine animals such as killer whale (Croft et al., 2016) where menopause occurs in that female albatrosses reproduce throughout their life until old ages. Why are males suffering a higher mortality at old ages than females? In terms of foraging, breeding males progressively shift their foraging zone to the south up to remote Antarctic waters, whereas young and middle‐aged males never foraged south of the Polar Front (Lecomte et al., 2010; Weimerskirch et al., 2014). Old males travelled a greater distance but were less active at the sea surface, and returned from sea with elevated levels of stress hormone (corticosterone), suggesting a low foraging efficiency (Lecomte et al., 2010). In parallel, the mass of old males progressively decreases after 30 years of age (Figure 3). Foraging efficiency of males appears to play a central role in shaping ageing patterns in natural conditions in wandering albatrosses, whereas females do not show a similar deleterious effect of ageing.

Personality is heritable (Patrick et al., 2013) and affected breeding success differently between males and females at old ages. Bold males had a higher reproductive success in later life than shy males, demonstrating a slower rate of senescence, whereas no such effects were found in females (Patrick & Weimerskirch, 2015). This is in accordance with the observation that during old ages bolder males gain more mass during foraging trips, suggesting they are able to replenish resources essential for successful reproduction. Bolder birds also make longer foraging trips as they get older. Such interaction between boldness and age did not occur at younger ages, nor for determining the age at first reproduction, indicating that these results are not driven by differences in early life. Thus, trade‐offs between current and future reproduction for bolder individuals may be exhibited in albatross but only during later life when reproductive success is more variable and has a stronger impact on survival (Patrick & Weimerskirch, 2015).

Thus, throughout their lifetime, our studies on wandering albatrosses have shown that foraging efficiency and the regulation of mass are the major factor regulating the trade‐off between breeding and survival (Figure 1). The mass‐dependent demography is likely to strongly influence the population dynamics, possibly by providing a delayed response in population size to environmental changes, generating autocorrelation in population fluctuations. Thus, to properly describe the population dynamics of such species, it would be important to develop in the future structured population models that include not only age but also body mass as a stage.

Many demographic parameters are influenced by environmental conditions at various stages of the life cycle of the wandering albatrosses. In particular, wind conditions play a central role for the foraging efficiency of albatrosses (Pennycuick, 1982; Weimerskirch, Guionnet et al., 2000) and it is no surprise that environmental conditions strongly affect demography through foraging and allocation processes (Figure 1). Climate change has affected wind in the Southern Ocean, with westerly winds having increased in intensity and moved pole ward (Thompson & Solomon, 2002; Weimerskirch, Louzao, de Grissac, & Delord, 2012). As a consequence, over the past 20 years, the foraging range of wandering albatrosses has shifted to the south in conjunction with these changes in wind pattern (Péron et al., 2010), while their rates of travel and flight speeds had increased (Weimerskirch et al., 2012). Consequently, the duration of foraging trips has decreased, breeding success has improved, and birds have increased in mass by more than one kilogram. These positive consequences of climate change may be temporary if patterns of wind in the southern westerlies follow predicted climate change scenarios suggesting a further shift towards south of strong wind area. Again these results based on long‐term records stress the importance of foraging performance as the key link between environmental changes and population processes.

## 5 DEMOGRAPHY AND FORAGING ECOLOGY AS TOOLS FOR CONSERVATION

Understanding the patterns and processes that generate changes in population size over time is essential for the conservation of species and their conservation status such as for IUCN listing. Yet, this is often not sufficient to understand the ultimate causes of a decline in survival or breeding success which is the source of the decline. Long‐term studies of animals can not only help us to answer important questions in ecology, but also play a key role in conservation. The case of the wandering albatross monitoring programme on Crozet is a striking example. Long‐term monitoring of the Crozet population detected as soon as the late 1980s that wandering albatrosses had declined severely over the past 20 years (Weimerskirch & Jouventin, 1987), similarly to the South Georgia population (Croxall, 1979). However, whereas at South Georgia, only population censuses were considered, and the causes of the decline were not known, the study at Crozet indicated that the decline of the population was due to a decrease in female adult survival. The decline was likely due to fishing operation in the range of females that favoured warmer waters than males (Weimerskirch & Jouventin, 1987). From this first study suggesting—possibly long lining as a potential threat because hooks were founds in the colony at Crozet, the first dedicated observations on board Japanese long liners confirmed that long lining for tuna was killing large numbers of albatrosses and petrels (Brothers, 1991). The Crozet study indicated that mortality probably occurred during breeding, but tuna long lining was located at more than 1,000 km from Crozet, a distance that was not considered at this time to be within the foraging range for a breeding seabird. In 1989, the first tracks of wandering albatrosses proved that breeding albatrosses had foraging range of more than 2,000 km (Jouventin & Weimerskirch, 1990), and following studies at Crozet showed that mainly breeding females from Crozet were able to reach the fishing zones of tuna long liners in subtropical waters while breeding (Weimerskirch, Salamolard, Sarrazin, & Jouventin, 1993). The first study combining tracks of foraging albatrosses with detailed distribution year after year of Japanese long liners in the Southern Ocean showed that the Crozet population overlapped with the Japanese long‐line fishery and that the development of this fishery coincided with the decline of wandering albatrosses at Crozet, but also in other populations of the species (Weimerskirch et al., 1997). After these first results, many studies have been carried out throughout the Southern Ocean and have shown that the long‐line fisheries constitute the major threat for albatross and petrels world‐wide (Croxall et al., 2012), and conservation programmes have been developed to reduce by catch, with good success in several fisheries, although the problem remains for other fisheries operating in international waters. Thus, the Crozet long‐term monitoring programme, and the associated tracking of birds from the same population, was crucial for detecting the cause of the decline of wandering albatrosses and the origin of the problem, long‐line fisheries operating at thousands of kilometres from the colonies. The shift towards the south of females due to changes in wind regime related to climate change could reduce in the future the interactions between wandering albatrosses and tuna long‐line fisheries (Weimerskirch et al., 2012).

Today fisheries are spread to the north of the range of wandering albatrosses, and the use of Radar detectors fitted on birds with GPS indicates that nearly 80% of individuals from Crozet encounter boats during their foraging trips (Weimerskirch et al., 2017). Such a widespread and almost systematic attendance to boats suggests that the foraging behaviour of wandering albatrosses has been modified over the past 50 years when fisheries have spread in the Southern Ocean. Thus, not only long‐line fisheries have increased mortality rates of all categories of the population, but the presence of boats has induced changes in the foraging routines of individuals (Collet, Patrick, & Weimerskirch, 2017). Historic analyses of albatross–fisheries interactions suggest that birds may not have modified their foraging zones but rather fisheries occupy the original foraging zone of the species (Weimerskirch, 1998). It will be important in the future to understand why wandering albatrosses are attracted since historical time by boats, and measure the benefits of attending boats to balance them with costs.

## 6 CONCLUSIONS

During the recent years, there is an increasing number of studies aiming at understanding the links between foraging and demography, especially linking habitat selection to demography (Matthiopoulos et al., 2015), and more rarely integrating body condition in the link between habitat selection and demography, for example (McLoughlin et al., 2007). The Crozet wandering albatross population is probably the one of the few wild population where demography, allocation and foraging behaviour have been studies simultaneously, with a few case studies in terrestrial habitats (Clutton‐Brock, Guinness, & Albon, 1982; Grant & Grant, 2002). In addition, an important by‐product of the combined studies is that the results had unexpected important applications in terms of conservation, as it is the study that allowed to detect the conservation problem of albatrosses linked to fisheries. This result alone stresses the importance of long‐term studies as sentinels of the long‐term changes occurring in the environment.

The long‐term study of the Crozet wandering albatross population has been successful in tackling several questions that have not been addressed similarly so far in other animal systems. They mainly concern the links between foraging, mass (allocation) and demographic parameters, with strong interactions between sex and age. In the wandering albatross, sexes differ in size, and mass affects multiple interrelated physiological, behavioural and ecological processes. The larger size of males confers them higher travel speed in windier conditions and higher fasting abilities but also higher energetic requirements. These advantages may be operating during some stages of their lifetime, at adulthood, and favour, for example, the migratory behaviour of males or their preference for windier southern conditions, but may be disadvantageous at others, that is during the juvenile and old age stages as suggested by the higher mortality rates at these stages. This example illustrates the complexity of interactions occurring across a life‐history strategy that has evolved over thousands of years.

## DATA ACCESSIBILITY

This synthesis does not use new data.
